# The need for protection: a cross-sectional analysis of direct exposure to firearm violence, attitudes towards firearms for protection, and firearm acquisition in California and Louisiana

**DOI:** 10.1186/s40621-026-00670-9

**Published:** 2026-03-16

**Authors:** Julia M. Fleckman, Lexie Contreras, Andrea DaViera, Priyanka Patel, Yingwei Yang, Jakana Thomas, Jennifer Wagman, Anita Raj

**Affiliations:** 1 Washington University in Saint Louis, 1 Brookings Drive, St. Louis, MO 63130 USA; 2 Tulane University, 1440 Canal St, New Orleans, LA 70112 USA; 3 University of Alabama, 729 Colonial Dr, Tuscaloosa, AL 35401 USA; 4 University of California San Diego, 9500 Gilman Dr., CA 92093 La Jolla, USA; 5 University of California Los Angeles, 650 Charles E Young Dr S, Los Angeles, CA 90095 USA

**Keywords:** Firearm violence, Violence exposure, Firearm acquisition, Firearm attitudes, Violence prevention, Perceived safety

## Abstract

**Background:**

Firearm violence is a leading cause of injury and death in the United States. Understanding how exposure to firearm violence shapes attitudes and behaviors related to firearms is critical for informing prevention and intervention strategies. This study examined associations between recent and lifetime firearm violence exposure and attitudes toward firearms for safety, as well as recent firearm acquisition. We hypothesized that recent and direct firearm violence exposure would be associated with increased firearm acquisition, whereas direct exposure recently and across the lifetime would be associated with more positive attitudes toward firearm ownership for protection.

**Methods:**

A cross-sectional study was conducted using data from the 2023 Violence Experiences (VEX) surveys, state-representative, population-based online surveys of adults 18 and older in California (CA) and Louisiana (LA). Data was combined from both states (*n* = 4492). Exposure variables included recent (past 12 months) and lifetime direct firearm violence exposure. Outcomes included attitudes toward having a firearm in the home for safety and past-year firearm acquisition. Poisson regressions with robust standard errors were utilized to examine the associations between violence exposure and attitudes toward firearms, and multinomial regressions to examine the association between violence exposure and firearm acquisition.

**Results:**

Respondents from LA reported higher lifetime exposure to violence, more positive attitudes toward having a firearm for safety, and greater recent firearm acquisition. Lifetime firearm violence exposure was associated with increased odds of positive attitudes regarding firearms for safety (APR 1.46, 95% CI 1.22–1.74), while recent exposure was associated with increased odds of firearm acquisition in the past year (ARR 4.15, 95% CI 2.36–7.28). Individuals with lifetime exposure had 1.50 the odds of reporting recent firearm acquisition compared to those with no lifetime exposure (95% CI 1.11, 2.03). Similar trends were found for state-specific analyses related to attitudes and acquisition.

**Conclusions:**

Findings suggest that prior direct exposure to firearm violence may be associated with both attitudes toward firearms and decisions to acquire them. Future research should explore how state-level policies may mitigate these effects and identify strategies to support individuals who acquire firearms for protection following exposure to violence.

**Supplementary Information:**

The online version contains supplementary material available at 10.1186/s40621-026-00670-9.

## Background

Firearm injury and mortality are a significant public health concern in the U.S. Firearm-related deaths are among the top three causes of death for individuals aged 1–44 [1]. Further, there were over 171,000 firearm-related emergency room visits in 2022 [1]. Beyond high incidence, both firearm injuries and deaths have significant adverse social, psychological, and economic impacts [2–6].

In recent years, research has increasingly focused on the motivations for firearm ownership, with particular emphasis on exposure to firearm violence. In 2023, nearly 79% of firearm owners cited protection and perceived safety as primary motivations for their firearm ownership [6]. However, protection may be deeply influenced by individuals’ exposure to firearm violence [8, 9], which shapes both their decisions to own and carry firearms.

Exposure to violence influences firearm carriage and ownership [7–13], often motivated by perceived safety needs. Individuals with direct exposure to violence (e.g., personal victimization and witnessing firsthand) are more likely to report owning a firearm for protection compared to individuals without exposure [9, 11]. Direct exposure to violence may be particularly important to consider for intervention due to increased risk for negative long-term effects, including increased likelihood of revictimization and involvement with the criminal justice system [14, 15].

Rates of firearm morbidity and mortality, as well as firearm regulations and policies vary widely by state. California (CA) and Louisiana (LA) are two states on opposite ends of the spectrum for firearm-related mortality and regulations. CA has the strongest firearm laws in the country and some of the lowest rates of firearm violence nationally, while LA experiences higher-than-average rates of firearm mortality and weak firearm-related regulations [16, 17]. Furthermore, as of 2016, LA’s average percentage of adults living in a household with a firearm was more than 2.5 times that of CA (48% compared to 18%) [18]. As of 2018, roughly one in four Californians had access to firearms in their home and 14% personally owned a firearm [19]. Also as of 2018, more than half of handgun owners (56.7%) in CA cite protection as their primary reason for ownership [20]. Conversely in LA as of 2023, where 42% of residents report personally owning a firearm, 44% endorse the belief that keeping a firearm in or around the home could help keep one safe [21].

Despite clear evidence that exposure to violence can impact decisions around firearm carrying and ownership, there is limited research examining how firearm violence exposure may be associated with firearm acquisition or purchasing, with a particular focus on two states with differing firearm landscapes, CA and LA. The aim of this study is to examine the associations between recent and lifetime direct firearm violence exposure and attitudes related to firearm ownership for protection, as well as exposure and recent firearm acquisition in CA and LA. We hypothesize that more recent firearm victimization is associated with more recent firearm acquisition, and that any lifetime firearm victimization is associated with more positive attitudes related to having a firearm for safety and protection. A deeper understanding of these relationships, and how they may vary between CA and LA, may help inform programs and policies related to improving firearm safety with the goal of reducing firearm injuries and mortalities.

## Methods

We analyzed cross-sectional data from the 2023 Violence Experiences (VEX) surveys conducted in CA and LA [22, 23]. VEX surveys, implemented by the National Opinion Research Center (NORCIn) at the University of Chicago, were state-representative online surveys designed to examine lifetime experiences of various forms of violence, as well as demographic and attitudinal characteristics. Surveys targeted adults aged 18 and above, utilizing both probability-based and non-probability-based online panels to meet sample size requirements for each state. NORC’s AmeriSpeak Panel is a probability-based panel designed to be representative of the U.S. household population (coverage of 97% of households) [24]. Randomly selected households are sampled using area probability and address-based sampling, with sampled households receiving outreach via mail, telephone, and field interviewers. This panel was additionally supplemented with respondents from non-probability online opt-in panels. Weighting was applied and TrueNorth statistical calibration was conducted by NORC to combine probability and non-probability samples [25]. Responses for the CA VEX survey (CalVEX) were collected between March and May 2023 (*n* = 3560), and for the LA Vex survey (LaVEX) between May and June 2023 (*n* = 1081). NORC sent email and phone reminders to panellists every four days throughout the fielding period. Participation rates were 21% for CalVEX and 27% for LaVEX, aligning with NORC’s standards for online surveys to produce population-based estimates. Additional details on sampling methods and participation rates are available in respective CalVEX and LaVEX 2023 reports [26, 27]. All study procedures received Institutional Review Board approval from Tulane University. Participants completed the survey in approximately 15 min and were compensated with the cash equivalent of $4.00. The current study included data from both surveys (*N* = 4,641).

### Measures

#### Independent variables

Firearm violence exposure was examined utilizing two survey questions. Proximal, or more recent exposure, was measured with the item: ‘Someone threatened or hurt you with a gun within the past 12 months,’ with responses recorded as *Yes/No*. This item and an additional item (‘Someone threatened or hurt you with a gun over 12 months ago’) were combined to create a lifetime exposure variable with *Yes/No* responses.

#### Dependent variables

Two dependent variables were examined. Attitudes toward firearms related to protection were assessed through a single item: ‘Having a firearm in or around your home can help keep your home and family safe.’ Response options included *Yes/No*. Recent firearm acquisition was assessed with the item: ‘Have you obtained a firearm in the *past 12 months?*’ Responses were initially recorded as: ‘Yes, I have obtained a firearm for the first time’, ‘Yes, this is not my first firearm’, ‘No, but I have a firearm’, and ‘No, and I do not have a firearm at this time’. This variable was then categorized as: *Yes* (for both Yes response options), *No but owns a firearm*, and *No does not own a firearm*. Belief and acquisition measures were utilized in prior versions of the CalVex survey [26, 27]; however, items were not formally validated.

#### Covariates

Several factors were included as potential confounders based on their established association with exposure to firearm violence, firearm attitudes, and firearm acquisition. Sociodemographic factors included: age, annual household income, race, ethnicity, employment, state of residence, population density for residence (Rural/Urban), education, and gender. Age was categorized (18–29, 30–44, 45–59, and 60 or older), as well as annual household income ($30,000 or less, $30,000-$60,000, $60,000-$100,000, and more than $100,000). Metropolitan vs. non-metropolitan designations were defined using the Office of Management and Budget criteria for metropolitan and micropolitan statistical areas [28]. Designations are county based and metropolitan includes counties or groups of counties with at least one urbanized area of 50,000 people or more and adjacent counties with economic ties to this central area. Non-metropolitan areas must include at least one urban area with a population of 10,000 to 49,999. Race/ethnicity was self-reported and measured using a single, combined race/ethnicity variable provided by NORC as part of the VEX survey data. Response options were “non-Hispanic white”, “non-Hispanic Black”, “non-Hispanic Asian”, “Hispanic”, and “Other/multiple races”. Educational attainment was categorized as “less than high school”, “high school”, “some college”, and “college degree”. Gender was defined as “male” and “female”.

### Analysis

Univariate, bivariate, and multivariate analyses were performed using Stata 17. Weighted descriptive statistics were first calculated for all study variables overall and stratified by state. Differences between California and Louisiana were assessed using design-based Pearson F statistics that accounted for the complex survey design. Then we conducted a series of Poisson regressions with robust standard errors to examine the associations between independent variables firearms violence exposure (recent and lifetime exposure) and the dependent variable of attitudes toward firearms for protection, given that this outcome is quite common [29]. Results from these models are presented as prevalence ratios (PRs) and 95% confidence intervals. Finally, we conducted a series of multinomial regressions to examine the association between independent variables firearms violence exposure (recent and lifetime exposure) and the dependent variable of firearm acquisition. Results from these models are presented as relative risk ratios (RRRs) with 95% confidence intervals. P-values of less than 0.05 were considered statistically significant for all analyses. Analyses were weighted to yield population estimates, and pooled analyses combining respondents from California and Louisiana were conducted to increase statistical power and estimate overall associations across states. State-specific models are presented for the associations between lifetime exposure and attitudes toward firearms and recent firearm acquisition. Due to small sample sizes for recent exposure to violence (CA: 1.89%, LA: 1.20%), combined models including both states are presented for associations with attitudes and acquisition (see eTables 1–2 for exploratory state stratified results). State of residence was included as a covariate only in models that combined both states.

## Results

Sociodemographic characteristics of the sample are found in Table 1. Half of the sample identified as female (50.53%), and 47.87% as male. Less than 20% reported being 29 years of age or younger, with 29.96% between the ages of 30 and 44, 22.77% between the ages of 45 and 59, and 28.58% over the age of 60. Just under half of the sample identified as non-Hispanic White (39.32%) and 33.76% identified as Hispanic. Over half of the sample reported having either completed high school or some college (52.08%), and income levels varied, with 32.83% earning over $100,000 and 23.39% earning less than $30,000. Most participants (52.40%) worked full-time, and the majority (89.55%) resided in CA. A small proportion (1.27%) reported exposure to gun violence in the past year, with 12.75% reporting lifetime exposure. Respondents from LA notably had significantly more reported lifetime firearm violence exposure compared to CA (21.34% vs. 11.75%; f = 28.69; *p* < 0.001). Additionally, more respondents in LA (43.60%) reported positive attitudes about having a firearm in the home for safety compared to those in CA (31.89%) (f = 22.95; *p* < 0.001). Finally, more respondents from LA reported acquiring a firearm in the past year compared to those in CA (10.08% vs. 5.02%; f = 4.03; *p* < 0.001).

**Table 1:** Sample characteristics (*N* = 4641)

	Total Sample			California			Louisiana		
	unwt (*N*)	wt (%)	95% CI	unwt (*N*)	wt (%)	95% CI	unwt (*N*)	wt (%)	95% CI
**Gun Violence Exposure Past Year**									
No	4,539	98.73	[98.06, 99.16]	3514	98.80	[98.03, 99.27]	1,043	98.10	[97.22, 98.71]
Yes	79	1.27	[0.84, 1.94]	46	1.20	[0.73, 1.97]	33	1.90	[1.29, 2.78]
Lifetime Gun Violence Exposure*									
No	3,950	87.25	[85.75, 88.61]	3088	88.25	[86.63, 89.69]	880	78.66	[74.73, 82.13]
Yes	668	12.75	[11.39, 14.25]	472	11.75	[10.31, 13.37]	196	21.34	[17.87, 25.27]
Attitudes toward firearms for safety*									
No	3,044	66.88	[64.70, 68.99]	2357	68.11	[65.73, 70.40]	621	56.40	[52.01, 60.69]
Yes	1,597	33.12	[31.01, 35.30]	1137	31.89	[29.60, 34.27]	460	43.6	[39.31, 47.99]
Firearm acquisition in past year*									
Yes	356	5.55	[4.60, 6.69]	215	5.02	[4.00, 6.28]	141	10.08	[8.05, 12.56]
No but I own firearm	856	17.94	[16.31, 19.69]	574	16.31	[14.59, 18.19]	282	31.83	[27.75, 36.22]
No and I do not have a firearm	3,399	76.51	[74.57, 78.35]	2,746	78.67	[76.57, 80.63]	653	58.08	[53.69, 62.36]
**Age (Years)**									
18–29	1,009	18.70	[16.83, 20.72]	710	19.00	[16.95, 21.24]	299	16.11	[13.56, 19.03]
30–44	1,426	29.96	[27.89, 32.12]	1,076	30.41	[28.14, 32.78]	350	26.09	[22.61, 29.89]
45–59	961	22.77	[20.87, 24.79]	738	22.69	[20.61, 24.90]	223	23.47	[19.93, 27.43]
60+	1,245	28.58	[26.61, 30.63]	1,036	27.90	[25.78, 30.13]	209	34.33	[30.09, 38.83]
**Income**									
Less than $30,000	1,348	23.39	[21.43, 25.46]	896	21.70	[19.58, 23.98)	452	37.86	[33.79, 42.10]
$30,000 to under $60,000	1,084	22.08	[20.16, 24.12)	807	21.96	[19.87, 24.21]	277	23.06	[19.72, 26.79]
$60,000 to under $100,000	949	21.71	[19.87, 23.67]	749	21.82	[19.81, 23.97]	200	20.76	[17.28, 24.72]
$100,000 or more	1,260	32.83	[30.74, 34.99]	1,108	34.52	[32.22, 36.90]	152	18.32	[15.02, 22.17]
**Race**									
White, Non-Hispanic	1,794	39.32	[37.17, 41.50]	1,159	36.64	[34.34, 39.00]	635	62.27	[57.98, 66.37]
Black, Non-Hispanic	1,058	7.77	[7.05, 8.55]	730	5.30	[4.72, 5.94]	328	28.91	[25.12, 33.02]
Asian, Non-Hispanic	645	14.93	[13.55, 16.42]	635	16.57	[15.02, 18.24]	10	0.89	[0.40, 1.96]
Hispanic	905	33.76	[31.31, 36.30]	839	37.14	[34.49, 39.88]	66	4.76	[3.27, 6.87]
Others	239	4.23	[3.48, 5.13]	197	4.35	[3.54, 5.34]	42	3.18	[1.99, 5.04]
**Employment**									
Full time	2,343	52.40	[50.09, 54.71]	1,788	52.96	[50.43, 55.49]	555	47.59	[43.27, 51.95]
Part time	531	19.94	[9.55, 12.50]	413	10.99	[9.47, 12.71]	118	10.52	[8.17, 13.47]
Not working	1,767	36.66	[34.48, 38.90]	1,359	36.05	[33.67, 38.50]	408	41.88	[37.63, 46.26]
**State**									
California	3,560	89.55	[88.56, 90.45]						
Louisiana	1,081	10.45	[9.55, 11.44]						
**Residence**									
Rural	325	2.81	[2.34, 3.37]	170	1.74	[1.33, 2.27]	155	11.97	[9.56, 14.90]
Urban	4,297	97.19	[96.63, 97.66]	3,377	98.26	[97.73, 98.67]	920	88.03	[85.10, 90.44]
**Education**									
Less than HS	279	11.24	[9.46, 13.31]	192	11.43	[9.47, 13.73]	87	9.64	[7.36, 12.52]
Completed HS/some college	2,519	52.08	[49.77, 54.39]	1,821	51.12	[48.58, 53.64]	698	60.36	[56.02, 64.54]
Completed bachelor’s	1,060	20.73	[19.14, 22.42]	886	21.19	[19.45, 23.04]	174	16.8	[13.84, 20.26]
Graduate degree	783	15.95	[14.59, 17.40]	661	16.27	[14.79, 17.86]	122	13.2	[10.45, 16.55]
**Gender**									
Woman	2,752	50.73	[48.40, 53.05]	2,013	50.22	[47.67, 52.77]	739	55.03	[50.58, 59.40]
Man	1,812	49.27	[46.95, 51.60]	1,483	49.78	[47.23, 52.33]	329	44.97	[40.60, 49.42]

* Significant differences (*p* < 0.05) between states using design-based Pearson F statistics that accounted for the complex survey design

There was no significant bivariate association between recent exposure to firearm violence and attitudes towards firearms safety and protection (PR 1.18, 95% CI 0.67, 2.05), but a significant and positive association between lifetime exposure to violence and attitudes was present (PR 1.39, 95% CI 1.19, 1.62). Adjusted models examining the associations between recent and lifetime exposure to firearm violence, and attitudes toward firearms related to safety for both states are presented in Table 2. In adjusted models, the association between recent exposure to firearm violence in the past year and attitudes regarding the utility of firearms in the home for safety was not significant. Lifetime exposure was significantly and positively associated with positive attitudes regarding the presence of firearms in the home for safety for both CA (APR 1.45, 95% CI 1.17–1.78) and LA (APR 1.49, 95% CI 1.06–2.09) respondents. Trends with key covariates largely mirrored those in the models with recent exposure and attitudes.

**Table 2:** Association between firearm violence exposure and positive attitudes toward firearms for protection

	Total Sample		Total Sample	California	Louisiana
	*N* = 4522		*N* = 4522	*N* = 3465	*N* = 1057
	APR [95% CI]		APR [95% CI]	APR [95% CI]	APR [95% CI]
**Gun Violence Exposure Past Year**		**Gun Violence Ever**			
(ref = No)		(ref = No)			
Yes	1.39 [0.86, 2.24]	Yes	1.46*** [1.22, 1.74]	1.45*** [1.17,1.78]	1.49* [1.06, 2.09]
**Age (Years)**		**Age (Years)**			
(ref = 18–29)		(ref = 18–29)			
30–44	0.97 [0.81, 1.17]	30–44	0.95 [0.79, 1.14]	1.03 [0.83,1.29]	0.78 [0.55, 1.09]
45–59	1.21 [0.99, 1.48]	45–59	1.18 [0.97, 1.45]	1.24 [0.97,1.58]	0.95 [0.64, 1.41]
60+	1.02 [0.82, 1.26]	60+	1.00 [0.81, 1.23]	1.04 [0.81,1.34]	0.93 [0.61, 1.42]
**Income**		**Income**			
(ref=Less than $30,000)		(ref=Less than $30,000)			
$30,000 to under $60,000	1.37* [1.14, 1.65]	$30,000 to under $60,000	1.38* [1.15, 1.66]	1.18 [0.95,1.47]	1.78* [1.26, 2.50]
$60,000 to under $100,000	1.41* [1.16, 1.71]	$60,000 to under $100,000	1.43* [1.18, 1.74]	1.30* [1.03,1.64]	1.59* [1.09, 2.31]
$100,000 or more	1.55* [1.27, 1.90]	$100,000 or more	1.57* [1.28, 1.93]	1.50* [1.18,1.89]	1.68* [1.07, 2.63]
**Race**		**Race**			
(ref=White, Non-Hispanic)		(ref=White, Non-Hispanic)			
Black, Non-Hispanic	0.80* [0.68, 0.95]	Black, Non-Hispanic	0.80* [0.67, 0.95]	1.03 [0.83,1.28]	0.51* [0.38, 0.69]
Asian, Non-Hispanic	0.68* [0.55, 0.85]	Asian, Non-Hispanic	0.70* [0.56, 0.86]	0.79* [0.63,1.00]	0.50 [0.12, 0.2.08]
Hispanic	0.64* [0.52, 0.78]	Hispanic	0.63* [0.52, 0.77]	0.72** [0.58,0.91]	0.41* [0.23, 0.73]
Others	1.08 [0.81, 1.45]	Others	1.06 [0.79, 1.42]	1.22 [0.88,1.70]	0.88 [0.45, 1.70]
**Employment**		**Employment**			
(ref=Full time)		(ref=Full time)			
Part time	1.00 [0.81, 1.23]	Part time	0.98 [0.79, 1.20]	1.07 [0.85,1.36]	0.70 [0.45, 1.09]
Not working	1.05 [0.90, 1.23]	Not working	1.05 [0.90, 1.22]	1.04 [0.87,1.25]	1.07 [0.79, 1.46]
**State**		**State**			
(ref=California)		(ref=California)			
Louisiana	1.43* [1.22, 1.68]	Louisiana	1.42* [1.21, 1.67]		
**Residence**		**Residence**			
(ref=Rural)		(ref=Rural)			
Urban	0.66* [0.52, 0.83]	Urban	0.66* [0.52, 0.85]	0.51* [0.36,0.71]	0.8 [0.55, 1.14]
**Education**		**Education**			
(ref= Less than HS)		(ref = < High School)			
Completed HS/Some college	1.62* [1.21, 2.17]	High School	1.59* [1.18, 2.13]	1.55* [1.09,2.22]	1.61 [0.95, 2.73]
Bachelor’s degree	1.22 [0.89, 1.69]	College	1.21 [0.88, 1.67]	1.21 [0.88,1.67]	1.72 [0.93, 3.18]
Graduate degree	0.94 [0.67, 1.33]	Graduate degree	0.93 [0.66, 1.32]	0.93 [0.66,1.32]	1.48 [0.77, 2.86]
**Gender**		**Gender**			
(ref=Female)		(ref=Female)			
Male	1.36* [1.19, 1.55]	Male	1.32*** [1.16,1.50]	1.32* [1.16,1.50]	0.89 [0.67, 1.19]

Abbreviations: APR, adjusted prevalence ratio; CI, confidence interval

*p < 0.05

Crude associations between recent and lifetime exposure and firearm acquisition are presented in Table 3. There was a positive and significant crude association between recent exposure to firearm violence and recent firearm acquisition both within the past 12 months (RR 7.46, 95% CI 4.48, 12.40), but no significant association between only previous firearm acquisition (over 12 months ago) and recent firearm exposure (RR 1.62, 95% CI 0.89, 2.97). There were significant, positive associations for lifetime exposure and both recent (RR 1.87, 95% CI 1.41, 2.46) and previous (RR 1.88, 95% CI 1.55, 2.28) firearm acquisition. Results for CA and LA mirrored those of pulled results for both states (see Table 3).

**Table 3:** Unadjusted associations between firearm violence exposure and firearm acquisition

	Total Sample		California		Louisiana	
	*N* = 4492		*N* = 3440		*N* = 1052	
	**Yes**	No but I own firearm	**Yes**	No but I own firearm	**Yes**	No but I own firearm
(ref = No and I do not have a firearm)						
	**URR [95% CI]**	**URR (95% CI)**	**URR (95% CI)**		**URR (95% CI)**	**URR (95% CI)**
**Gun Violence Exposure Past Year**						
(ref = No)						
Yes	7.46* [4.48,12.40]	1.62 [0.89,2.97]	7.00*[3.53,13.90]	1.54 [0.69,3.43]	5.96* [2.69, 13.20]	1.36 [0.53,3.49]
**Lifetime Gun Violence Exposure**						
(ref = No)						
Yes	1.87* [1.41, 2.46]	1.88* [1.55, 2.28]	1.75* [1.22, 2.52]	1.86* [1.47, 2.36]	1.80* [1.15, 2.81]	1.74* [1.22, 2.47]

Abbreviations: URRR, unadjusted relative risk ratio; CI, confidence interval

*p < 0.05

Adjusted multivariate results for the association between exposure to firearm violence and recent firearm acquisition for both states are presented in Table 4. After adjusting for key covariates, the positive association between recent firearm violence exposure and recent firearm acquisition remained significant (ARR 4.15, 95% CI 2.36–7.28), although the confidence interval was wide, suggesting some imprecision in the estimate. Of note, the association between having a firearm that was acquired previously (more than a year) and recent violence exposure was not significant (ARR 1.62, 95% CI 0.89–2.97). There was no significant association between lifetime exposure and recent firearm acquisition for CA respondents (ARR 1.45, 95% CI 0.99–2.14), but CA respondents with lifetime exposure were 1.62 times more likely to have a firearm that was previously acquired (more than a year) compared to those with no lifetime exposure, after adjusting for key covariates (95% CI 1.25, 2.09). Similar results were seen for LA respondents, with no significant association between lifetime exposure to violence and recent firearm acquisition (AOR 1.53, 95% CI 0.93–2.50). However, participants from LA with lifetime exposure were 1.67 times more likely to have previously acquired a firearm (more than a year ago) compared to participants with no lifetime exposure (95% CI 1.14–2.46).

**Table 4:** Adjusted Associations Between Firearm Victimization and Firearm Acquisition

	Total Sample				Total Sample			California		Louisiana	
	*N* = 4492				*N* = 4492			*N* = 3440		*N* = 1052	
	**Yes**	No but I own firearm			**Yes**		No but I own firearm	**Yes**	No but I own firearm	**Yes**	No but I own firearm
(ref = No and I do not have a firearm)											
	**ARRR 95% [CI]**				**ARRR 95% [CI]**			**ARRR 95% [CI]**		**ARRR 95% [CI]**	
**Gun Violence Exposure Past Year**			**Lifetime Gun Violence Exposure**								
(ref = No)			(ref = No)								
Yes	4.15* [2.36,7.28]	1.53 [0.78,3.00]	Yes	1.50* [1.11,2.03]		1.65* [1.33,2.04]		1.45 [0.99,2.14]	1.62* [1.25,2.09]	1.53 [0.93,2.50]	1.67* [1.14,2.46]
**Age (Years)**			**Age (Years)**								
(ref = 18–29)			(ref = 18–29)								
30–44	0.67* [0.51,0.88]	1.39* [1.06,1.82]	30–44		0.64* [0.49,0.84]		1.36* [1.04,1.78]	0.60* [0.42,0.86]	1.63* [1.13,2.35]	0.75 [0.49,1.17]	1.17 [0.76,1.78]
45–59	0.38* [0.26,0.55]	1.66* [1.25,2.20]	45–59		0.36* [0.25,0.52]		1.60* [1.20,2.13]	0.36* [0.23,0.58]	1.97* [1.35,2.88]	0.38* [0.20,0.71]	1.27 [0.80,2.02]
60+	0.18* [0.11,0.29]	2.18* [1.64,2.91]	60+		0.17* [0.10,0.27]		2.13* [1.60,2.85]	0.17* [0.09,0.30]	2.61* [1.79,3.81]	0.15* [0.06,0.37]	1.57 [0.96,2.54]
**Income**			**Income**								
(ref=Less than $30,000)			(ref=Less than $30,000)								
$30,000 to under $60,000	1.09 [0.78,1.52]	1.37* [1.07,1.77]	$30,000 to under $60,000		1.12 [0.80,1.56]		1.38* [1.07,1.78]	0.98 [0.63,1.51]	1.45* [1.05,2.00]	1.23 [0.73,2.07]	1.11 [0.73,1.68]
$60,000 to under $100,000	1.38 [0.97,1.98]	2.39* [1.86,3.08]	$60,000 to under $100,000		1.43 [1.00,2.04]		2.45* [1.90,3.15]	1.19 [0.75,1.88]	2.26* [1.65,3.12]	1.82* [1.02,3.24]	2.57* [1.67,3.94]
$100,000 or more	1.50* [1.02,2.19]	2.69* [2.06,3.51]	$100,000 or more		1.57* [1.08,2.30]		2.75* [2.11,3.59]	1.43 [0.90,2.27]	2.52* [1.83,3.48]	1.9 [0.96,3.77]	3.41* [2.06,5.65]

Note: The reference outcome category is **“No**,** and I do not have a firearm**”

Abbreviations: ARRR, adjusted relative risk ratio; CI, confidence interval

*p < 0.05

## Discussion

We sought to address a gap in the research literature by examining the association between firearm violence exposure and both attitudes related to needing firearms for safety or protection and recent firearm acquisition across two distinctive geographic and policy contexts. Associations varied by timing of exposure. Lifetime firearm violence exposure was consistently associated with more favorable attitudes toward firearms for safety, whereas recent exposure was more strongly associated with recent firearm acquisition. Such findings are relevant to addressing threats to both public health and safety, particularly in highlighting the need for future exploration of exposure to violence across states with different policy environments and for designing interventions to reduce and address exposure to violence as well as firearm acquisition and ownership.

Our findings on the association between firearm violence exposure and firearm attitudes related to safety are mixed. Across the total sample, there was no significant relationship between recent exposure and more positively held attitudes about the need for a firearm in the home for safety, but we found a positive and significant association between lifetime exposure and positive attitudes. Findings build on previous studies examining the link between both direct and indirect exposure to violence and the fear of being victimized [11] as well as beliefs in having access to firearms for safety [30]. It may be that individuals who have been exposed to violence do not immediately have positive firearm attitudes related to safety, and instead those develop over time, or that our analysis was underpowered. However, additional research is warranted to examine this relationship further. Additionally, recent research has broadened the conceptualization of violence exposure to include witnessing shootings, knowing someone who has been shot, and being threatened with a firearm [31, 32]. Given that our assessment of direct firearm violence exposure (including being threatened or hurt) may underestimate the full spectrum of exposure for individuals, incorporating broader experiences of both direct and indirect exposure in future work is critical for assessing how each may shape firearm attitudes and acquisition.

Additionally, we found that individuals with recent exposure to violence had higher odds for reporting acquisition of a firearm in the past year for all respondents compared to individuals without recent exposure. This finding builds on past research connecting both direct and indirect violence exposure and an increased risk for firearm carriage [7, 10] and ownership [8, 9, 13]. However, no studies, to our knowledge, have examined the direct association between exposure to violence and firearm acquisition. Similar to potential motivations for firearm carrying after exposure to firearm violence, individuals who have experienced violence may feel a need to acquire a firearm for fear of their own safety or protection. Additional research is needed to specifically examine motivations for firearm acquisition after exposure to violence, as some studies have highlighted gun behaviors may be more episodic in nature, with risk for gun carrying happening closer to when individuals are exposed to violence, particularly for young adults [7, 10, 33, 34]. Given that firearm acquisition may be related to more episodic exposure to violence, our study sought to examine how recent and chronic exposure were associated with acquisition. However, given a small sample of individuals who reported distal exposure (before the last 12 months), we were unable to assess this. Future studies should parse out whether acquisition differs between individuals with recent-only exposure, distal-only exposure, and chronic exposure. Additionally, more indirect forms of violence (e.g., hearing gunshots) should be examined.

Despite CA and LA being very different states, there are similar patterns of findings across certain models. For example, after adjusting for sociodemographic covariates, lifetime gun violence exposure was positively related to attitudes toward firearms in the home for the entire sample and CA and LA individually. However, it is notable that there are state-level differences in both our exposure and outcome variables. LA had significantly higher levels of lifetime gun violence exposure (11.75% in CA compared to 21.34% in LA), more positive attitudes (31.89% in CA opposed to 43.6% in LA), and greater rates of ownership (in CA, 76.57% of surveyed residents did not own any firearm, while only 53.69% of LA residents did not own a firearm). This may potentially have to do with differences between the two states’ cultural and policy environments. It may be that LA residents have more positive beliefs toward firearms in the home because violence is more prevalent and firearm regulations are more relaxed [16, 17, 21]. The implications of this may be a greater perception of the need for firearms to protect one’s safety in the home.

The substantially different firearm policy environments and cultures in LA and CA may contribute to differences in participants’ exposure to violence, attitudes toward firearms, and acquisition patterns. Future research is needed with larger, multi-state samples to systematically examine how differing policy and firearm cultural environments, as seen in these two states, influence firearm-related attitudes and behaviors. Although past research has shown that older adults are more likely to have pro-gun beliefs related to safety [35], youth and young adults who have been exposed to firearm violence and to firearms in general are more likely to display pro-gun beliefs and a range of risky firearm related behaviors, relative to the same aged people without such exposure [10, 36]. Further, while not directly assessed in this study, prior work suggests that gender norms, rural identity, and income may all play a role in shaping pro-firearm beliefs and beliefs about safety and protection [37, 38] as well as gun ownership [39]. Future research should explore these mechanisms.

Existing firearm injury efforts have primarily focused on safe storage training and education, rather than motivations for firearm acquisition. Evidence suggests that counseling combined with education on locking devices can increase safe storage behaviors, particularly when delivered in healthcare or community settings [40]. However, such approaches typically assume firearm ownership and often do not engage with individuals perceived safety needs or the circumstances surrounding firearm acquisition. Another emerging approach to reducing firearm violence is the community violence intervention model, which focuses on conflict mediation, case management support, and mentorship delivered via credible messengers with lived experience [41]. Given that violence exposure may increase firearm carriage [42] and based on our findings, acquisition, educational and supportive interventions should consider ways to address individuals’ perceptions around needing a firearm for protection or safety that may result from fear while also promoting ways to mitigate risk for additional injury including reducing access, and safe storage of firearms. Additionally, the psychological impacts of violence exposure including PTSD must be addressed, given that they are intertwined with feelings of fear and a disrupted sense of safety [43], and potentially the need to acquire a firearm.

The current study is not without its limitations. First, the cross-sectional design limits our ability to assess causal relationships between exposure to firearm violence, beliefs about firearms in the home, and recent firearm acquisition. Longitudinal research is needed to more clearly parse out temporal and causal pathways. Further, the use of non-validated measures for the key outcome and exposures, as well as-self-reported survey data raise the potential for measurement error, including social desirability bias, recall bias, and misclassification of firearm violence exposure, beliefs, and acquisition. However, the survey was anonymous and self-administered, which may have reduced the potential for social desirability bias and underreporting of exposure to violence and firearm acquisition. Further, measures utilized for direct firearm violence exposure, beliefs, and acquisition have been used across multiple rounds of data collection and were pilot tested to ensure comprehension. There is also low missingness of data, indicating potential validity. Additionally, given that recent exposure to violence was a rarer exposure, we were underpowered to examine state specific associations with attitudes and firearm acquisition. Future research is warranted to examine such associations with a larger sample size to assess state differences. The survey also did not collect geographic identifiers, such as zip code, which limited our ability to link responses to external data sources and examine potentially relevant neighborhood level factors such as community violence rates. Future research should explore individual and neighborhood level variables that may relate to gun violence beliefs and acquisition, such as individual political affiliation, and neighborhood level poverty and crime. Additionally, although models adjusted for state and stratified analyses yielded similar results, unmeasured contextual differences between California and Louisiana may remain and could influence the generalizability of pooled estimates. Another limitation is that because both firearm violence exposure and firearm acquisition were assessed over the same 12-month period, we cannot determine whether firearm acquisition preceded or followed firearm violence exposure within that timeframe. Finally, although study results may not be generalizable to a broader national population, the focus on two states with distinct firearm policies and norms is novel and provides important insight into how state-level contexts may shape firearm-related attitudes, behaviors, and exposure to violence.

## Conclusions

This study aimed to illuminate how exposure to gun violence plays a role in positive beliefs about firearms and firearm acquisition across CA and LA, two different state policy environments. Our analysis found that lifetime exposure to gun violence was significantly positively associated with positive attitudes toward firearms for safety, and both recent and lifetime exposure to gun violence were significantly positively related to recent and lifetime firearm acquisition respectively. These findings suggest that those who have positive attitudes toward having firearms and acquire firearms may feel a “need for protection,” considering their previous exposure to violence. Thus, interventions aiming to reduce firearm acquisition should consider ways to validate this need, along with ways to mitigate risk for firearm owners including education regarding firearm safety, access, and safe storage as well as provision of safe storage devices [40]. Additionally, the significant relationships that we observed account for state-specific differences but indicate that future research should consider how state-level gun policy and rates of local gun violence may influence individuals’ perceived need to carry a firearm and their beliefs about firearms.

## Electronic Supplementary Material

Below is the link to the electronic supplementary material.

Supplementary Material 1

## Data Availability

The data used in this study are restricted and cannot be shared.

## Abbreviations

VEX: Violence Experiences
CA: California
LA: Louisiana
NORC: National Opinion Research Center
CalVEX: California violence experiences
LaVEX: Louisiana violence experiences
