# Supplementary material for: The need for protection: a cross-sectional analysis of direct exposure to firearm violence, attitudes towards firearms for protection, and firearm acquisition in California and Louisiana
Source: Inj Epidemiol. 2026 Mar 16;13:29. doi: 10.1186/s40621-026-00670-9 (PMC13104192; doi:10.1186/s40621-026-00670-9)
Supplement: Supplementary file 1 — Supplementary Material 1 [file 40621_2026_670_MOESM1_ESM.docx]

**eTable 1. Association Between Recent Firearm Victimization and Positive Attitudes toward Firearms for Protection**

|  | **California** | **Louisiana** |
| --- | --- | --- |
|  | **N=3483** | **N= 1062** |
|  | **AOR 95% CI** | **AOR 95% CI** |
| **Gun Violence Past Year** |  |  |
| (ref=No) |  |  |
| Yes | 1.80* [1.02,3.48] | 0.96 [0.43, 2.12] |
| **Age (Years)** |  |  |
| (ref=18-29) |  |  |
| 30-44 | 1.05 [0.84, 1.31] | 0.81 [0.58, 1.14] |
| 45-59 | 1.27 [1.00, 1.62] | 1 [0.68, 1.47] |
| 60+ | 1.07 [0.83. 1.38] | 0.98 [0.65, 1.48] |
| **Income** |  |  |
| (ref=Less than $30,000) |  |  |
| $30,000 to under $60,000 | 1.17 [0.94, 1.46] | 1.77*** [1.26, 2.49] |
| $60,000 to under $100,000 | 1.29* [1.02, 1.62] | 1.56* [1.07, 2.26] |
| $100,000 or more | 1.47** [1.17, 1.86] | 1.68* [1.08, 2.63] |
| **Race** |  |  |
| (ref=White, Non-Hispanic) |  |  |
| Black, Non-Hispanic | 1.04 [0.84,1.29] | 0.52* [0.38, 0.70] |
| Asian, Non-Hispanic | 0.79* [0.63,0.99] | 0.48 [0.12, 2.00] |
| Hispanic | 0.72** [0.58,0.90] | 0.42** [0.24, 0.75] |
| Others | 1.25 [0.90,1.74] | 0.85 [0.44, 1.64] |
| **Employment** |  |  |
| (ref=Full time) |  |  |
| Part time | 1.09 [0.86,1.39] | 0.72 [0.46, 1.11] |
| Not working | 1.05 [0.87,1.25] | 1.07 [0.79, 1.45] |
| **Residence** |  |  |
| (ref=Rural) |  |  |
| Urban | 0.49*** [0.35,0.68] | 0.8 [0.56, 1.15] |
| **Education** |  |  |
| (ref=Less than HS) |  |  |
| HS/Some college | HS1.58* [1.11,2.25] | 1.66 [0.99, 2.81] |
| Bachelor’s degree | 1.1 [0.75,1.61] | 1.74 [0.95, 3.20] |
| Graduate degree | 0.81 [0.54,1.22] | 1.49 [0.77, 2.86] |
| **Gender** |  |  |
| (ref=Female) |  |  |
| Male | 0.93 [0.70, 1.23] | 1.57*** [1.35, 1.83] |
| Abbreviations: AOR, adjusted odds ratio; CI, confidence interval  **p* <0.05 | | |

**eTable 2. Association Between Recent Firearm Victimization and Firearm Acquisition in the Past Year**

|  | **California** | | **Louisiana** | |
| --- | --- | --- | --- | --- |
|  | Yes | No but I own firearm | Yes | No but I own firearm |
|  | (ref=No and I do not have a firearm) | | | |
|  | **ARR 95% CI** | | **ARR 95% CI** | |
| **Gun Violence Past Year** |  |  |  |  |
| (ref=No) |  |  |  |  |
| Yes | 3.90* [1.84,8.29] | 1.58 [0.66,3.81] | 3.99* [1.64,9.68] | 1.42 [0.49,4.13] |
| **Age (Years)** |  |  |  |  |
| (ref=18-29) |  |  |  |  |
| 30-44 | 0.63* [0.44,0.90] | 1.67* [1.16,2.40] | 0.79 [0.51,1.23] | 1.21 [0.79,1.84] |
| 45-59 | 0.38* [0.24,0.61] | 2.02* [1.39,2.96] | 0.42* [0.22,0.78] | 1.34 [0.84,2.12] |
| 60+ | 0.18* [0.10,0.33] | 2.66* [1.82,3.89] | 0.16* [0.06,0.40] | 1.62 [1.00,2.62] |
| **Income** |  |  |  |  |
| (ref=Less than $30,000) |  |  |  |  |
| $30,000 to under $60,000 | 0.95 [0.62,1.48] | 1.43* [1.04,1.97] | 1.19 [0.70,2.02] | 1.11 [0.73,1.68] |
| $60,000 to under $100,000 | 1.17 [0.73,1.85] | 2.21* [1.61,3.05] | 1.73 [0.96,3.10] | 2.48* [1.62,3.81] |
| $100,000 or more | 1.38 [0.87,2.19] | 2.47* [1.79,3.40] | 1.76 [0.88,3.52] | 3.34* [2.02,5.53] |
| **Race** |  |  |  |  |
| (ref=White, Non-Hispanic) |  |  |  |  |
| Black, Non-Hispanic | 0.58* [0.36,0.95] | 0.59* [0.45,0.78] | 1.3 [0.84,2.01] | 0.52* [0.36,0.77] |
| Asian, Non-Hispanic | 0.58* [0.36,0.92] | 0.41* [0.30,0.56] | 1.15 [0.13,10.46] | 0.59 [0.11,3.05] |
| Hispanic | 0.79 [0.52,1.20] | 0.57* [0.42,0.76] | 1.12 [0.53,2.37] | 0.79 [0.41,1.52] |
| Others | 1.09 [0.57,2.09] | 0.95 [0.62,1.45] | 0.92 [0.30,2.83] | 1.15 [0.55,2.42] |
| **Employment** |  |  |  |  |
| (ref=Full time) |  |  |  |  |
| Part time | 1.3 [0.85,1.98] | 1.36 [1.00,1.85] | 0.74 [0.38,1.42] | 1.19 [0.73,1.94] |
| Not working | 0.62* [0.42,0.93] | 1.08 [0.85,1.37] | 0.68 [0.41,1.11] | 0.88 [0.62,1.27] |
| **Residence** |  |  |  |  |
| (ref=Non-Metro) |  |  |  |  |
| Metro | 0.52* [0.27,0.99] | 0.49* [0.33,0.72] | 0.69 [0.41,1.18] | 0.71 [0.47,1.08] |
| **Education** |  |  |  |  |
| (ref=<HS) |  |  |  |  |
| Completed HS/some college | 1.06 [0.67,1.68] | 1.75* [1.32,2.33] | 0.73 [0.38,1.43] | 1.03 [0.63,1.70] |
| Bachelor's degree | 0.74 [0.46,1.19] | 1.26 [0.94,1.68] | 1.09 [0.52,2.27] | 1.07 [0.61,1.85] |
| Graduate degree | 0.88 [0.44,1.78] | 0.59 [0.34,1.04] | 1.46 [0.56,3.84] | 1.01 [0.48,2.14] |
| **Gender** |  |  |  |  |
| (ref=Female) |  |  |  |  |
| Male | 3.10* [2.28,4.22] | 1.82* [1.49,2.20] | 2.22* [1.48,3.33] | 1.50* [1.09,2.08] |

Note: The reference outcome category is **"No, and I do not have a firearm**".

Abbreviations: ARRR, adjusted relative risk ratio; CI, confidence interval

**p* <0.05
